# A Synthetic Method for Atmospheric Diffusion Simulation and Environmental Impact Assessment of Accidental Pollution in the Chemical Industry in a WEBGIS Context

**DOI:** 10.3390/ijerph110909238

**Published:** 2014-09-05

**Authors:** Haochen Ni, Yikang Rui, Jiechen Wang, Liang Cheng

**Affiliations:** 1 Jiangsu Provincial Key Laboratory of Geographic Information Science and Technology, Nanjing University, Nanjing 210093, China; E-Mails: haochenni.nju@gmail.com (H.N.); ruiyikang@gmail.com (Y.R.); lcheng@nju.edu.cn (L.C.); 2 Department of Geographic Information Science, Nanjing University, Nanjing 210093, China

**Keywords:** air pollution, chemical accident numeric simulation, environmental impact, emergency decision making

## Abstract

The chemical industry poses a potential security risk to factory personnel and neighboring residents. In order to mitigate prospective damage, a synthetic method must be developed for an emergency response. With the development of environmental numeric simulation models, model integration methods, and modern information technology, many Decision Support Systems (DSSs) have been established. However, existing systems still have limitations, in terms of synthetic simulation and network interoperation. In order to resolve these limitations, the matured simulation model for chemical accidents was integrated into the WEB Geographic Information System (WEBGIS) platform. The complete workflow of the emergency response, including raw data (meteorology information, and accident information) management, numeric simulation of different kinds of accidents, environmental impact assessments, and representation of the simulation results were achieved. This allowed comprehensive and real-time simulation of acute accidents in the chemical industry. The main contribution of this paper is that an organizational mechanism of the model set, based on the accident type and pollutant substance; a scheduling mechanism for the parallel processing of multi-accident-type, multi-accident-substance, and multi-simulation-model; and finally a presentation method for scalar and vector data on the web browser on the integration of a WEB Geographic Information System (WEBGIS) platform. The outcomes demonstrated that this method could provide effective support for deciding emergency responses of acute chemical accidents.

## 1. Introduction

The chemical industry has deeply reformed many aspects of our modern daily lives. It has also become fundamental in other domains, such as transportation, medicine, the manufacturing industry, and the high-tech industry. The consumption of chemical products can be enormous, especially in highly populated and developed areas. Owing to transportation costs, and the resulting agglomeration effects, chemical production is usually located in developed coastal areas and around population centers [1]. The continuous production of hazardous and toxic chemicals ultimately necessitates the storage of the products in the manufacturing facilities, which could potentially present a security risk to industry personnel and the neighboring population [2]. In the event of an accident in the production chain, hazardous material may be released into the atmosphere instantaneously. As a result, it would cause tremendous harm to the eco-environment and public health [3].

In order to mitigate the damage of a chemical accident, a great deal of research has focused on the statistical analysis of accident risk [2] and executive emergency response plans [4,5]. With the development of environmental numeric simulation models [6,7,8,9] and model integration techniques [10,11], many Decision Support Systems (DSSs) have been established to help manage chemical hazards [12,13,14,15,16,17]. A recent survey found that most DSS are based on quantitative risk assessments of potential accident sources and used to predict and prevent major accidents. According to the process of risk management, they can be classified into five categories: political or strategic decision-making; prioritization decision-making; identification decision-making; analysis decision-making; and evaluation decision-making [12]. Only a small amount of research utilizes DSSs for acute accident simulation and emergency responses. However, limited by the technical capacity and design idea, these DSSs always have shortcomings in some respects. First the majority of DSSs can only handle specific types of accidents, mostly hazard release and diffusion [13,15,16]. Few take explosion and conflagration into consideration, but due to the serial simulation strategy, cannot realize the multi-type simulation simultaneously [14,17]. Most of aforementioned DSSs lack the function of comprehensive environmental impact assessment. Second, other DSSs have utilized real-time data processing (including meteorology data and accident source information) [6,13], but cannot present real-time simulation results. Third, the system architecture for DSS is mostly in the client/server mode [13,14,15,16,18]. This category of system architecture is easy to design and implement, although the software modification for system function amendments, at client and servers ends, are very involved. Additionally, the client/server architecture cannot be interoperated by multi-users in network environments.

In order to improve the system inadequacies, an organized mechanism of model sets based on the accident type and pollutant substance is proposed, along with a scheduling mechanism for parallel processing of multi-accident-type, multi-accident-substance, and multi-simulation-models; and a presentation method for scalar and vector data on the web browser. In consideration of spatial data management, organization, expression, and network interoperation, the WEB Geographic Information System (WEBGIS) is selected as the integration platform. Geography Information System (GIS) is an important tool to integrate and manage environmental modelling [19] and WEBGIS is a cross-platform integration framework, which has good functional scalability and suitable performance in spatial representation [20]. Other novel technologies, such as .NET cross-platform framework, WKT data format, and the FLEX expression technique, were also employed [21,22]. Using matured atmospheric simulation models and the novel techniques discussed, the complete workflow of the emergency response was established, including raw data (meteorology information, and accident information) management, numeric simulation of different types of accidents (leakage, explosion, conflagration), environmental impact assessments, and representation of simulation results. The outcomes demonstrated the comprehensive and real-time simulation of acute accidents in the chemical industry.

In Section 2 the comprehensive modelling of acute accident in chemical industry is presented, and Section 3 demonstrates the integration method of the environment models in the WEBGIS platform. At last, some application examples are displayed in Section 4 to validate the method proposed in this paper.

## 2. Numerical Simulation of the Accidental Air Pollution in the Chemical Industry

A comprehensive investigation was carried out in a nutritional feed additives company, the Nanjing Chemistry Industry Plant. The information of the potential risk source (include storage amount, component, state, and spatial location of the hazard substance) and historical accidents in the factory was systematically collected. The investigation of the factory and the statistical research of acute accidents in the chemical industry [1,3] concluded that acute accidents could be divided into three operational objectives, according to the object-oriented design mode. These are leakage, conflagration, and explosion [23,24]. The characteristics of each accident were used to adopt the mature numerical simulation model to acquire the function of the three operational components.

### 2.1. Leakage

Leakage is the most common accident in the chemical industry [2,25]. Once toxic and harmful substances leak into the atmosphere, they will cause tremendous harm to the eco-environment. When the substance concentrations surpass a threshold value, more serious accidents may occur, such as conflagration and explosion. Hence, leakage modes and substance diffusion patterns have been researched for many years.

In the study of atmospheric pollutant diffusion simulation, a number of models have been devised with distinctive application features. Among the most widely applied models are the hybrid model SLAB (Atmospheric Dispersion Model for Denser than Air Releases) developed by Lawrence Livermore National Laboratory of U.S. Department of Energy [26], and the DEGADIS (Dense Gas Atmospheric Dispersion) developed by the U.S. Coast Guard and Gas Research Institute [7]. This paper uses SLAB as the core code to simulate the leakage of substances in the atmospheric environment. This model has been tested by several simulation studies and is widely applied to a number of practical applications [7,26]. Using specific cloud profile functions in two- or three-dimensional processing of conservation equations, the SLAB model applies the 3D spatial diffusion to one-dimensional shallow model, only relevant to downwind distance or time. The process is based on the 3D conservation equations of mass, momentum, energy, and composition, which change with time, in combination the ideal gas equation and cloud morphological equations. The SLAB model is efficient and has good compatibility, which can support multiple types of leakage mode, including ground pool evaporation, above-ground level ejection, upward ejection, instantaneous diffusion, and short-time ground pool evaporation. These characteristics meet the demand for urgent responses to sudden chemical accidents [27].

A total of 27 characteristic parameters were selected to drive the SLAB model adopted, in accordance with the application features of the chemical industry. These parameters can be distributed into five leakage type categories (represented by code numbers). These include the physicochemical properties of the leakage substance, status parameter, regional parameter, and meteorological conditions (Table 1). Chlorine was selected as the input example; Table 1 details the parameters defined.

**Table 1:** Input parameter of the Atmospheric Dispersion Model for Denser than Air Releases (SLAB) model, for the chlorine example. The pink text indicates the leakage type; the red text represents the physicochemical properties of leakage substance; the blue text is the leakage status parameters; the black text is the meteorological conditions; and the yellow text signifies the regional parameters.

Parameter	Value	Parameter	Value
Leakage Type (%idspl)	1	Height of source (%hs)	0
Molecular weight of source air (%wms)	0.0709	Concentration of average time (%tav)	30
Heat capacity in the fixed pressure (%cps)	499.1	Largest distance calculation (%xffm)	20000
Boiling point temperature (%tbp)	239.1	Height of the concentration calculated (%zp)	1/0/0/0
Evaporation heat consumption (%dhe)	287840	Initial water content (%cmed0)	0.88
Liquid heat capacity (%cpsl)	926.3	Source temperature (%ts)	239.1
Liquid density of source gas (%rhosl)	1574	Release intensity of source (%qs)	10
Saturated vapor pressure parameter (%spb)	1978.34	Source area (%as)	0.02
Unsaturated vapor pressure parameter (%spc)	-27.01	Release duration (%tsd)	1200
Environmental height — stations (%za)	10	Relative position x of source (%xx)	326.7
Environment temperature (%ta)	293.16	Relative position y of source (%yy)	758.1
Relative humidity (%rh)	70	Surface roughness (%z0)	S
Stability(%stab)	4		
Wind direction (%wd)	90		
Environmental wind speed (%ua)	4		

### 2.2. Conflagration

Conflagration is another type of sudden accident that triggers extensive damage to the eco-environment, and especially human life. These accidents are usually initiated by the mixing of leaked inflammable gas or liquids and ignition sources. The main detriment of the conflagration is thermal radiation. The temperature of fire accident sites was simulated according to heat generation rates using the function:

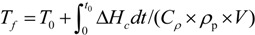
(1)
here, Δ*H_c_* is combustion enthalpy (Δ*H*/*kJ*/*mol*) (Table 2), *C_p_* is the specific heat capacity (*J*/(*kg·K*)), *ρ*_p_ is the space density and *V* is the space volume (*m*^3^).

**Table 2:** Combustion enthalpy of typical inflammable substances in the chemical industry.

Name	Chemical Formula	ΔH (kJ/mol)	Name	Chemical Formula	ΔH (kJ/mol)
Hydrogen	H_2_(g)	−285.8	Ethane	C_2_H_6_(g)	−1559.8
Carbon monoxide	CO(g)	−283.0	Ethylene	C_2_H_4_(g)	−1411.0
Methane	CH_4_(g)	−890.31	Acetylene	C_2_H_2_(g)	−1299.6
Methanol	CH_3_OH(l)	−726.51	Ethanol	C_2_H_5_OH(l)	−1366.8
Benzene	C_6_H_6_(l)	−3267.5	Propane	C_3_H_8_(g)	−2219.9

### 2.3. Explosions

Accidents involving explosions in the chemical industry are defined as physical or chemical, depending on the origin [28]. These two kinds of explosions may have subtle distinctions in the action mechanisms, but both instigate huge calamity to the environment and human beings. In the chemical industry, sudden accidents, such as explosion, conflagration, and pollutant leakage, are always concomitant. It is of great importance to the emergency responses to estimate the influence of explosions accurately. The destructive power of explosions can be approximately quantified and simulated using three physical quantities: overpressure field, thermal radiation, and pollutant dispersion [29].

Gas overpressure, otherwise known as a shock wave, is the most destructive effect of the explosion. In the chemical industry, raw materials, intermediates, and final products are stored in pressurized vessels or pipelines. In addition, some materials are inflammable. The total energy of the explosion, which will stimulate the shock wave, consists of two parts: the energy of pressurized vessel and the gas explosion energy [30]. To understand the first part, the following equation may be applied:

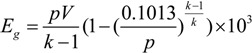
(2)
here, *E_g_* is the energy of the pressurized vessel (*kJ*), *p* is the absolute pressure of the vessel (*MPa)*, *V* is the volume of the vessel (*m*^3^), and *k* is the gas adiabatic index (the ratio of specific heat at constant pressure and specific heat at constant volume). The calculation of the second part is as follows:

*E_c_* = *C_ρ_* × *ρ*_p_ × *V* (3)

here, *E_c_* is the explosion energy of the gas (*kJ*), *C_p_* means specific heat capacity (*J*/(*kg·K*)), *ρ_p_* is the gas density (*kg*/*m_3_*), and *V* is the space volume (*m*^3^).

Previous research has shown that the strength of the overpressure is positively correlated to the cube root of the explosive power, and is negatively correlated to the distance from the explosion center [31], shown as:

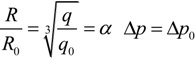
(4)
here, *R* is the distance from the explosion center *m*, *q_0_* means explosion energy generated by standard amount of TNT (Trinitrotoluene) (*kJ*); *q* means explosion energy generated by the actual explosion (*kJ*), *Δp* is the overpressure at the target (*MPa*), *Δp_0_* is the overpressure at the standard (*MPa*), and *α* is the ratio of the explosion experiment.

Assuming a 1000 kg TNT explosion, for which the explosion energy is approximately 4500 kJ, it was possible to simulate the overpressure field of different explosion source. Table 3 shows the overpressure values at the different distances from the explosion center. Table 4 shows the different influences to buildings and humans at the certain threshold values.

**Table 3:** Relationship between the shock wave overpressure and distance from a 1000 kg TNT explosion.

Distance/m	Overpressure/MPa	Distance/m	Overpressure/MPa	Distance /m	Overpressure/MPa
5	2.94	16	0.235	50	0.0235
6	2.06	18	0.170	55	0.0205
7	1.67	20	0.126	60	0.0180
8	1.27	25	0.079	65	0.0160
9	0.95	30	0.057	70	0.0143
10	0.76	35	0.043	75	0.0130
12	0.50	40	0.033		
14	0.33	45	0.027		

**Table 4:** Damage caused by shock waves to buildings and human health. The threshold values are adapted from a study by Fu *et al.* [32]. The symbol/denotes no influence is observed.

Serial Number	Overpressure/MPa	Damage to the Buildings	Damage to the Human Health
1	0.000–0.005	/	/
2	0.005–0.006	Partly broken doors and windows	/
3	0.006–0.015	Majority broken of doors and windows broken	/
4	0.015–0.020	Complete damage to doors and windows	/
5	0.020–0.030	Wall cracks	Slight injury
6	0.040–0.050	Serious wall cracks, roofing tile damage	Auditory organs injury or fracture
7	0.060 and above	Wood beams broken，room frames loose	Visceral serious injury or death

In addition to overpressure, chemical explosions ignited by the inflammable substances can release large quantities of energy and heat via thermal radiation. According to leaking velocity and combusting materials, different materials have different enthalpies (Table 2); the overall thermal radiation flux of fire source caused by the explosion can be determined [33]. Firstly, the thermal radiation value of the ignited source can be calculated using:

*q* = *ηQ*_0_*H_c_* (5)

here, *q* is the thermal radiation flux of point heat source (*W*), η is the efficiency factor expressed as 0.35, *Q_0_* is the leakage speed (*kg*/*s*); *H_c_* is the combustion enthalpy (*J*/*kg*).

Secondly, the thermal radiation value along the radiation radius is established using the equation:

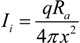
(6)
here, *I_i_* is the intensity of thermal radiation (*W*/*m^2^*), *q* is the radiation flux of the heat source (*W*), *R_a_* is the radiation rate with a default value of 0.2, *x* is the distance from the heat source *i* (*m*).

Chemical pollutant dispersion by explosions is very slight [34]. However, the hazards of the chemical substances necessitate the simulation of the dispersion of these pollutants. The dispersion of the explosive pollutants can be regarded as an instantaneous emission, thus, the Gaussian Puff Model can be applied to calculate the distribution mode of the pollutants [9]. The Gaussian Puff Model is expressed as:

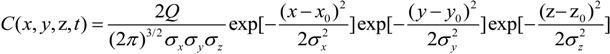
(7)
here, (*x*_0_, *y*_0_, *z*_0_) are the coordinates of the accident source, *C* is the puff ground concentration at the point (*x*, *y*, *z*) and moment *t_w_* (*mg*/*m*^3^), *Q* is the release amount of the puff (*mg*), *σ_x_σ_y_σ_z_* are the diffusion parameters in horizontal and vertical direction (*σ_x_* = *σ_y_* = *γ*_1_*t* , *σ_z_* = *γ*_2_*t*), *t* is the diffusion time (s), *γ*_1_ and *γ*_2_ are the regression coefficients of the horizontal and vertical diffusion parameters [35].

### 2.4. Environmental Impact Assessment

The selected models focus on the calculation of the physical quantities to simulation the common accidents in the chemical industry, but ignore the environmental influence of the hazardous chemical substance. Using the physical quantities simulated, an environmental impact assessment model was applied to evaluate the accident risk from the perspective of damage to life and health. In this study, the relatively mature acute exposure guideline levels (AEGLs) criteria [8] were chosen as the model for environmental influence assessment in a chemical emergency. Using the concentration values determined by simulation models, the damage areas were divided into four types: lethal, disabling, harming, and inhalation discomfort. Dangerous threshold values of different substances are shown in Table 5.

**Table 5:** The acute exposure guideline levels (AEGLs) threshold values of typical hazardous gas in the chemical industry.

Substance	AEGLs 0/ppm	AEGLs 1/ppm	AEGLs 2/ppm	AEGLs 3/ppm
CS_2_	2	17	200	600
NH_3_	3	30	220	2700
MSH	0.5	5	59	120
H_2_S	0.07	0.75	41	76
HCN	0.25	2.5	17	27
CH_3_OH	60	670	11,000	40,000
SO_2_	0.02	0.2	0.75	30
CH_2_CHCOOH	0.15	1.5	68	480
CH_2_O	0.09	0.9	14	100
C_2_H_3_CHO	0.003	0.03	0.44	6.2

## 3. The Organization Mechanisms of Model-Sets and Presentation of Simulation Results

In this paper, the research aim is to establish a synthetic method of atmospheric diffusion simulation and environmental impact assessment for emergency responses to accidental air pollution from the chemical industry. The simulation models described belong to different domains, with differing drive parameters and simulation. Simple assembly of these models would not suffice, especially given the widely spread web environment and access by multi-users. In order to meet the application demand, an integration mechanism was devised to amalgamate the simulation models. The integration mechanism can divide into three main parts: the organization pattern of the simulation models and calculated data, the scheduling scheme of the model set and data flow, and the representation mode of different numerical simulation results.

### 3.1. Organization Pattern of the Simulation Models and Calculated Data

Argent (2004) proposed four levels of model integration for development and application. The higher the level, the fewer demands and limitations the model application will have [10]. This paper applied various models in different domains, such as chemistry, environment, meteorology, and physics, in one project. The models differed in driven parameters, development operation environments, and formats of the results. Nevertheless, these models were also connected by data flow, with the results of one model driving the parameter of the other. The method used is unique because it is available on the web and accessible by novices and experienced users alike.

**Figure 1:**
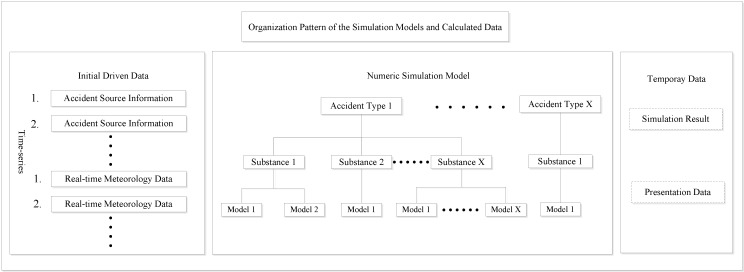
Conceptual design framework of the organization pattern of the simulation models and calculated data.

In order to get a higher level of integration, a specialized organization pattern must be designed for the simulation models and calculated data, the main design framework is shown in Figure 1. Considering the variety of pollutants, large amount of the calculated data (a long-term meteorology data and a wide cover of spatial numeric field data), and the need for scheduling simplicity, the models were organized on the basis of the pollutant substance and accident type. The models using the same pollutants, in a certain accident, are regarded as a single organizational unit. The organizational units for the same accident are aggregated to an application paradigm. It has small differences in driven parameters (namely the pollutant substance). These forms of organizational patterns require a great deal of storage space for the simulation models, but it will provide a tremendous advantage for reducing the scheduling complexity and post-maintenance workload. Hence, simple scheduling logic can be used to achieve the complex application task. Owing to the fact that the organizational units and simulation models are independent, changing the workflow for another application task can be realized by simply modifying part of the organizational unit and not the entire model set. Except for the simulation models, the initial driven (such as accident source information) and real-time meteorology data are stored in a time-series that guarantees the consistency of the accidental information and quick recall of the history accident record. The simulation results are temporary data, which only exist for data display on the terminal. The result data are usually in grid field format, which is it is very large, making it difficult to store continuously.

### 3.2. Scheduling Scheme of the Model-Set and Data Flow

**Figure 2:**
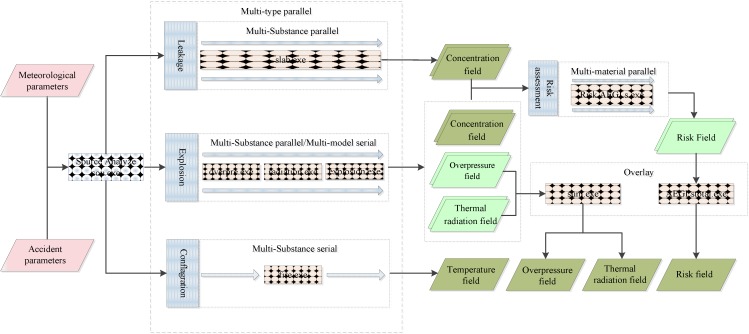
Flow chart of the model scheduling.

One of the prominent characteristics of the method is the cross-platform and network interoperability. When designing the scheduling scheme and data flow between the models, a special scheduling program was devised. The final scheme contained a two-level hierarchy scheduling program, made up of the upper scheduling module and the substrate model library. The upper scheduling module is based on the cross-platform framework .NET proposed by Microsoft. This framework has excellent compatibility with the lightweight server in the Microsoft Server platform, which is commercially available and widely used in engineering applications [22]. The upper scheduling module is coded in C#, and runs in the Microsoft .NET Framework 3.5 environment. It allows data exchange of the initial driven parameters and simulation results between the client and server sides, as well as the invocation and data flow of the model sets according to accident type and application demand. The substrate model library is the assembly of the simulation models. This two-level hierarchy scheduling strategy makes independence and cooperation possible between the scheduling program and substrate models. It achieves orderly concatenation of the multiple environmental impact models. The scheduling flow is shown in Figure 2. The model scheduling procedure consists of three main stages.

#### 3.2.1. Pollution Source Analysis

The driving parameter of this method is comprised of meteorological and accident parameters. The meteorological parameter contains wind direction, wind velocity, atmospheric temperature, and other common meteorological parameters. The accident parameter describes the accident type (leakage, explosion, or conflagration), accident substance species, storage of accident substance, and other information. The key fields decide the scheduling procedure. The sou.exe analyzes the parameter and transfers the relevant parameters to the simulation model, according to accident type and substance.

#### 3.2.2. Environmental Impact Simulation

This stage is the core of the scheduling procedure, which organizes the order of operation. The main scheduling strategy of this stage is the multi-type parallel that runs the simulation processes of the different accident types at the same time, in a synchronous or asynchronous way. This parallel operation mechanism is implemented by the simultaneous multi-threaded technology in the Net framework [36]. Different scheduling strategies were adopted according to the calculation procedure of the different accident types. For simulation processes where different pollutant substances are independent of each other, the calculation procedure will adopt the multi-material parallel strategy (such as the calculation procedure of leakage and explosion (Figure 2)). In contrast, relationships between the different substances, such as spatial overlay of the simulation result of atmospheric temperature in conflagration, will require the use of the multi-substance serial. This would mean the scheduling program will invocate the models individually in an organized order. Meanwhile, if the calculation procedure of one certain accident consists of more than one type of numeric simulation model, the strategy of multi-model serial is utilized. The simulation results may be the driven by the parameters of another model; hence, the scheduling program will invocate models according to the data flow among the simulation model sets, such as the calculation procedure for explosions (Figure 2).

#### 3.2.3. Processing of Simulation Results

The raw simulation results calculated by the models are often disordered and unorganized, with physical properties (such as concentration) of the same substance calculated by different models stored separately. Data management for final presentation at the user terminal is tedious. The representation characteristics of the web environment are low data transmission rate and high efficient interactive operation [37]. Therefore, spatial registration and overlay are conducted on the simulation results, before the processed data is presented at the web terminal.

### 3.3. Representation Mode of Different Numerical Simulation Results

Numerical simulation results can be divided into two categories, according to the spatial traits; these are scalar quantity data that only have magnitude (such as pollutant concentration, explosive overpressure, *etc.*) and vector data that have both magnitude and direction (such as wind direction). These two categories of data have different features in the presentation mode. Considering the limitations of data transmission through the web environment, a distinguishing method was included to achieve spatial expression at the client terminal.

**Figure 3:**
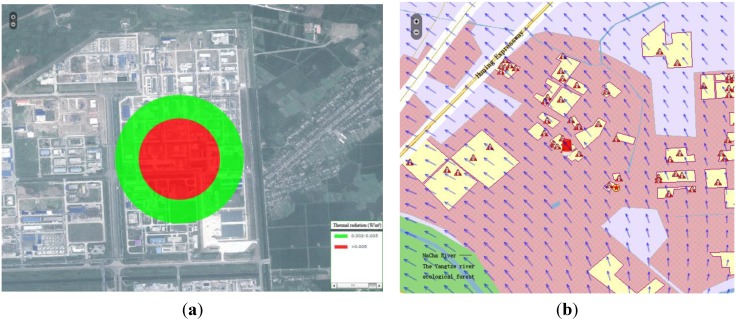
Spatial representation of the scalar and vector data in the context of the geo-reference environment. Image (**a**) shows the thermal radio field in the RS (Remote Sensing) image. Image (**b**) shows the wind field in the vector map.

The scalar data are usually organized in the regular grid data format, which has a simple structure but high data redundancy. It is difficult to establish rapid data transmission through the Internet, and the data cannot be utilized directly for data rendering by the WEBGIS client. An iso-surface generation method was implemented to the convert the gird data into several iso-surfaces, according to certain criteria, such as AEGLs and other security standards in the chemistry industry. This action is performed at the server end; hence, the client will only receive several iso-surfaces instead of the whole grid value. This reduces data transmission costs from the server to the client, and allows raster-vector conversion from regular grid data to vector presentation data. Figure 3a shows that the quantitative value usually decreases along the radial direction of the generation source. In order to eliminate this among the iso-surfaces, concentric circles were adopted as the presentation format for the iso-surface. The generation process of the concentric iso-surface can be described in three steps: firstly, sparse grids field data is condensed by the use of a spatial interpolation method; secondly, iso-lines are tracked by means of an iso-line generation algorithm; and thirdly, iso-lines of the same value and generate iso-surfaces between two iso-lines that have minimum interval value are amalgamated.

The wind field data is the most representative and important vector data for this research. Wind direction and velocity have a huge influence on pollutant dispersion (Figure 3b). Considering the spatial traits of the wind field, an arrowhead map method was devised for wind field expression. This method takes the arrow direction and length to represent wind direction and velocity, allowing the conversion of the amorphous wind field to the directed arrows, which are regularly distributed among the entire data field. This conversion greatly helps the presentation of the wind field at the client terminal. The detail of the process was as follows: firstly, statistical analysis was conducted on the wind direction and velocity of the whole data field; secondly, the length of the arrowhead was standardized by the maximum and minimum values of wind velocity; and, thirdly, the length and angle of each wind arrow was calculated and passed to the client terminal.

**Figure 4:**
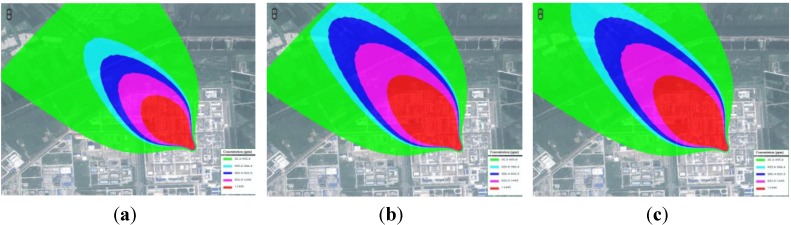
Time-series dynamic map for the leakage concentration field in the RS image (height 10 m). Images (**a**), (**b**), and (**c**) present the concentration field at the 5 min, 10 min, and 15 min, respectively, after the leakage accident.

Scalar and vector data were presented using the proposed method. It was found that the numeric simulation results cannot exclusively present the distribution features and variation tendencies in a dynamic and objective way. Additionally, the implications and environmental impact of the data must be put in context of the real geographical environment. Hence, WEBGIS was used as the presentation platform to express of the result data. WEBGIS provides a basic map service, which forms the basis map for the simulation data. Figure 3 shows the simulation results super-imposed on the vector map or raster image. The geography environment information around influenced area could then be easily obtained. In order to express the temporal-spatial variation of the simulation results, the time-series dynamic map was applied to the variation tendencies of result data (Figure 4). The dynamic map shows the evolution of the simulation process at several key time nodes.

Different simulation models usually select different data formats. It is necessary to unify the data format (arrowheads, concentric circles, and iso-surface) for better efficiency before representation at the client terminal. The data was organized in WKT format, as defined by OpenGIS. This is a text markup language for representing vector geometry objects on a map, spatial reference systems of spatial objects, and transformations between spatial reference systems [21]. It met the application requirements of GIS, and was also capable of spatial data interaction and rapid data transmission. The presentation technique at the client terminal was FLEX, as proposed by Adobe^®^, which is, globally, the most popular front-end display technology [38].

## 4. Application and Discussion

An application system was established for a nutritional feed additives company, the Nanjing Chemistry Industry Plant, which is one of the global leaders in nutritional feed additives with the utilization of the simulation models and organization mechanism presented in this paper. This system was deployed on the company intranet, providing the company officer emergency decision support for sudden chemical accidents. In order to test the method and validate the correction and feasibility of the application system, several trial accidents were constructed and used to simulate the environmental impact using the proposed method.

**Figure 5:**
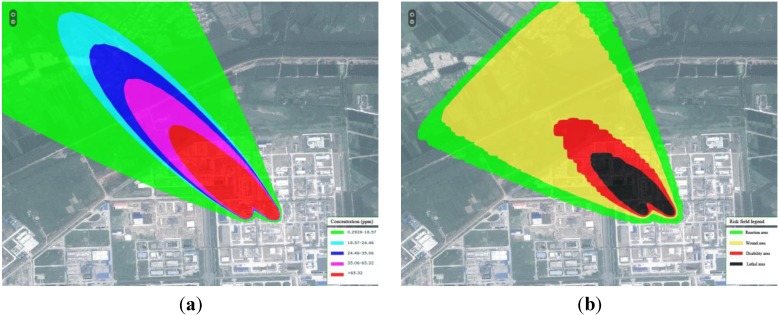
Gas-gas H_2_S leakage. Image (**a**) shows the concentration field and image, (**b**) shows the risk field.

**Figure 6:**
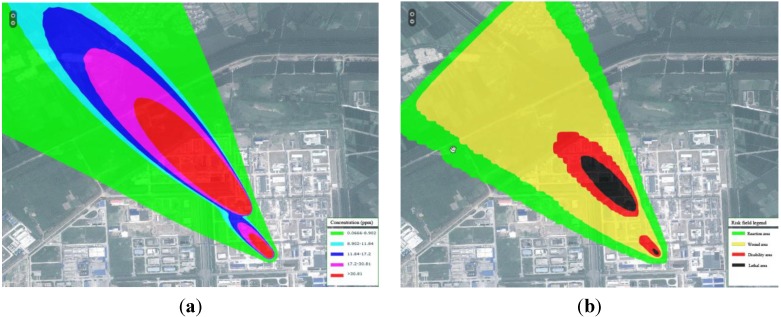
Gas-liquid H_2_S leakage. Image (**a**) shows the concentration field and image (**b**) shows the risk field.

As leakage is the most common accident in the chemical industry, two kind of multi-leakage accidents were conducted. Figure 5 shows the gas-gas H_2_S leakage. It was found that the two gas leakage sources amalgamate into a powerful entirety; the influences of the two sources were almost the same. Figure 6 shows the gas-liquid H_2_S leakage, the pollutant source in upper left (gas) is more intensive than the lower right (liquid). It was concluded that the gas leakage was more serious than the liquid leakage.

**Figure 7:**
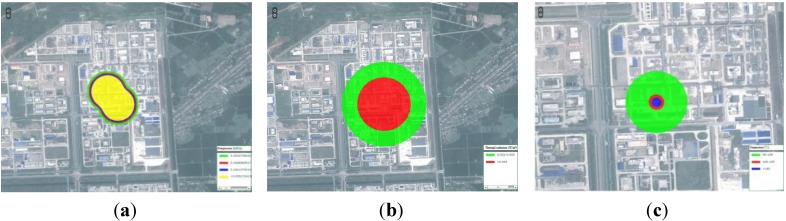
Simulation results of the explosion and conflagration. Images (**a**), (**b**), and (**c**) respectively present the overpressure, thermal radiation, and conflagration temperature. In (**a**), the overpressure of the upper left part represents the CS_2_ storage, and the lower part denotes H_2_S. In (**b**), the thermal radiation of the explosive shows that the combustion heart of CS_2_ is too low, thus, only the H_2_S thermal radiation is observed. In (**c**), the temperature of the CH_3_OH conflagration reaches 150 °C.

Explosions and conflagration are usually secondary accidents after leakage. Explosions often happen at the pressure vessel, and conflagration is caused by combustible substances igniting. Two pressure vessels stored liquid H_2_S and CS_2_ were chosen to simulate the influence of an explosion. A CH_3_OH leakage fire was used to estimate the conflagration effect (Figure 7).

The simulation results presented a typical accident in the chemical industry. The special organization patterns made the scheduling program and model sets independent from each other, meaning that a certain model of the model set could be replaced without the modification of the scheduling program and the addition of the new simulation procedure could be done with slight changes to the scheduling program. Using the .NET technique and the distinguished scheduling mechanism, the scheduling program applied the different scheduling strategies for various calculation processes and guided the data flow among the models in a cross-platform environment. The representation method for the scalar and vector data realized the dynamic and visible representation of the simulation results with a WEBGIS context. The synthetic method of atmospheric diffusion simulation and environmental impact assessment for emergency responses to accidental air pollution of the chemical industry proposed in this paper realizes the systemic integration of the various models and the efficiency of this synthetic method meets the demand of the actual application.

However, there remain limitations to the findings of this study. Firstly, the storage format of the model set is the file document, which is an efficient format when the quantity of models is small. A model database must be designed for the storage of the model sets. Secondly, although the method has been designed for the Internet environment, and is accessible to many users, the simulation calculations only respond to requests from one user. This means that only one user can use the simulation function at a time, but others can see the simulation results. The next development stage involved the application of parallel computing technology into the method, allowing multi-user concurrent operation. Thirdly, the simulation results are calculated by numerical models, the precision of the data must be improved through field monitoring data. Real-time sensors should be incorporated into the method to revise the simulation results.

## 5. Conclusions

Using field investigations and matured environmental simulation models, comprehensive modeling of acute accidents in the chemical industry (include leakage, conflagration, explosion, and environmental impact assessment) was conducted. The following were also devised: an organizational mechanism of the model set, based on the accident type and pollutant substance; a scheduling mechanism for the parallel processing of multi-accident-type, multi-accident-substance, and multi-simulation-model; and, finally, a presentation method for scalar and vector data on the web browser on the integration platform of WEBGIS. The workflow of the emergency response, including raw data (meteorology information, and accident information) management, numeric simulation of different kinds of accidents, environmental impact assessments, and representation of the simulation results were achieved. This allowed comprehensive and real-time simulation of acute accidents in the chemical industry. It was concluded that this method could provide effective support for deciding emergency responses in acute chemical accidents.
